# The role of fascin-1 in the pathogenesis, diagnosis and management of respiratory related cancers

**DOI:** 10.3389/fonc.2022.948110

**Published:** 2022-08-11

**Authors:** Naibin Zhang, Yankun Gao, Qiang Bian, Qianqian Wang, Ying Shi, Zhankui Zhao, Honglian Yu

**Affiliations:** ^1^ Department of biochemistry, Jining Medical University, Jining, China; ^2^ Collaborative Innovation Center, Jining Medical University, Jining, China; ^3^ Department of Pathophysiology, Weifang Medical University, Weifang, China; ^4^ The Affiliated Hospital of Jining Medical University, Jining Medical University, Jining, China

**Keywords:** fascin-1, respiratory related cancers, pathogenesis, diagnosis, biomarker, treatment

## Abstract

Human cancer statistics report that respiratory related cancers such as lung, laryngeal, oral and nasopharyngeal cancers account for a large proportion of tumors, and tumor metastasis remains the major reason for patient death. The metastasis of tumor cells requires actin cytoskeleton remodeling, in which fascin-1 plays an important role. Fascin-1 can cross-link F-actin microfilaments into bundles and form finger-like cell protrusions. Some studies have shown that fascin-1 is overexpressed in human tumors and is associated with tumor growth, migration and invasion. The role of fascin-1 in respiratory related cancers is not very clear. The main purpose of this study was to provide an updated literature review on the role of fascin-1 in the pathogenesis, diagnosis and management of respiratory related cancers. These studies suggested that fascin-1 can serve as an emerging biomarker and potential therapeutic target, and has attracted widespread attention.

## Introduction

Cancer is a major public health issue worldwide (1), and is also the leading cause of death in China and developed countries (2,3). Respiratory related cancers mainly include lung, laryngeal and nasopharyngeal cancers. According to the Annual Cancer Statistics Report 2022, respiratory related cancers account for a large proportion of all cancers (1). Compared with 2021 statistics, the number of lung cancer has no downward trend, and it is still the first cancer in mortality (4). Like most tumors, metastases are also the leading cause of death for patients with respiratory related cancers (5).

In order to achieve metastasis, cancer cells need to spread from the primary tumor to other organs and it will form a secondary tumor (6). At the same time, it is found that the remodeling of cytoskeleton is essential in the migration, invasion, and metastasis spread of cancer cells, in which actin plays a key role (7–10). The major class of actin that regulates these complex processes is fascin, which exists in humans and other vertebrates as fascin-1, fascin-2 and fascin-3, with fascin-1 being the most extensively studied (11). Fascin-1 is hardly expressed in normal human tissues. In contrast, high expression of fascin-1 has been found in a variety of cancers (12). Our previous study and many other studies revealed that fascin-1 can promote tumor cell migration, invasion, and metastasis (13). Our previous study and many other studies revealed that fascin-1 can promote tumor cell migration, invasion, and metastasis (13–16). A growing number of studies have shown that it can be used as a new biomarker and therapeutic target and to assess the prognosis of cancer patients. However, the mechanism of fascin-1 in respiratory related cancers is unclear.

In this review, we illuminate fascin-1 expression in respiratory related cancers and its partial mechanisms by discussing the recent literature dealt with the expression of fascin-1 expression in these cancers. Furthermore, we focus on the correlation between fascin-1 expression and clinicopathological parameters and its relationship with patient prognosis.

## Structure and function

**Figure 1:**
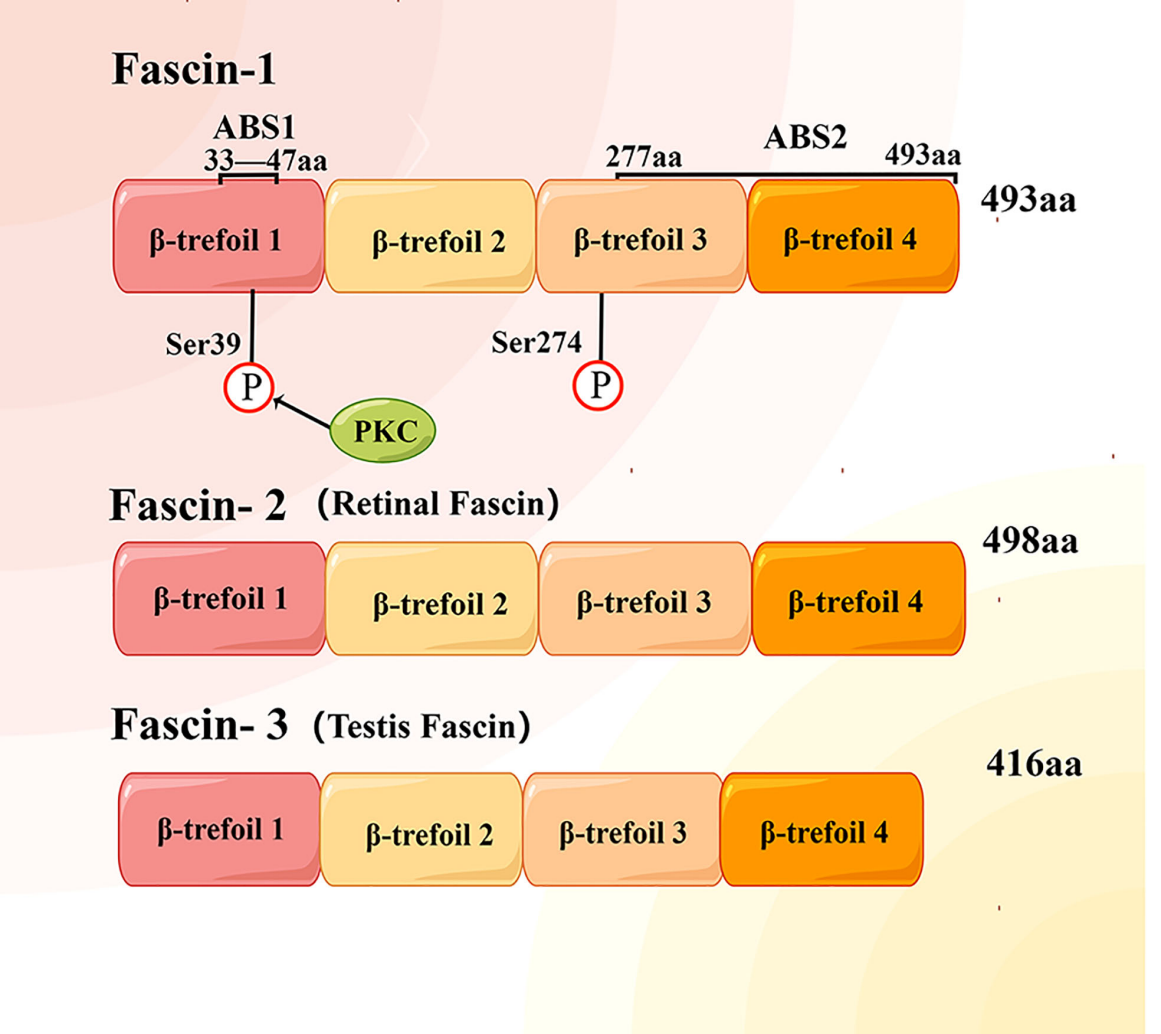
Structural diagram of human fascin protein family. Each fascin protein consists of four β-trefoil domains and with different molecular weight. Actin binding site 1 (ABS1) of fascin-1 protein is located between amino acids (aa) 33 and 47 of the first β-trefoil domain, while the location of ABS2 has not been determined.

Fascin is a globular protein with a size of about 55 KD, which is composed of four tandem fascin protein domains (17, 18) (**Figure 1**). Fascin-2 is mainly distributed in retinal photoreceptor cells (19), and fascin-3 is distributed in the head of spermatid cells (20). Structural studies have revealed that human fascin-1 protein consists of 493 amino acids, including four β-trefoil domains (21). One actin binding site (ABS) is located on aa33-47 in the β-trefoil 1 of fascin-1, but the second actin binding site has not been fully located (18, 22). At the ser-39 residue of the first binding site, it can be phosphorylated by highly conserved protein kinase C (PKC) (22–24). Ser274 phosphorylation can also regulate the actin binding ability of fascin-1 in human cancer cells (12). In addition, fascin-1 has been shown to interact with many proteins other than F-actin, such as MST2, TGF-β family type I, neurotrophins nerve growth factor (NGF) and neurotrophin-3 (NT-3) (25–29). Functional studies have shown that fascin-1 protein can promote the migration, invasion and metastasis of tumor cells (13, 15, 16). Fascin-1 also plays a role in diseases other than cancer, such as wound healing and neurological diseases. Therefore, it is necessary to further clarify the mechanisms of interaction between the fascin-1 protein and different proteins to understand these novel functions of fascin-1.

## Literature review

At present, nearly ten reviews on the relationship between fascin-1 and cancer have been published, showing that fascin-1 is overexpressed in a variety of cancers and its pathways of action and regulatory mechanisms have been partially elucidated (**Table 1**). Meanwhile, fascin-1 also plays a role in a variety of diseases other than cancer and in human embryogenesis.

**Table 1:** Main research contents and future prospects of literature review.

Author	Main content	Article Type	Future prospects	Ref.
Hashimoto et al.	Distribution of the three isoforms of fascin Fascin-1 expression and clinicopathological parameters of aggressive tumors Mechanism and results of fascin-1 upregulation	review	Overexpression of fascin-1 is expected to be a biomarker or therapeutic target	(9)
Liu et al.	Fascin-1 functions and their structures Biological effects of fascin-1 changes in vivo and in vitro experiments Expression of fascin-1 protein in tumor tissues Transcriptional regulation mechanisms and signaling pathways of fascin-1	review	Learn more about the function of fascin-1 in different human cancers and its related mechanisms	(21)
Hashimoto et al.	The fascin gene family and its evolution Distribution and role of the isoforms of fascin The role of fascin-1 and fascin-2 in different diseases	review	The potential of fascin-1 as a candidate target for tumor metastasis is increasing	(16)
Tan et al.	Correlation of fascin-1 with clinicopathological parameters in breast, colorectal, esophageal, gastric and lung cancers	Systematic review	Focusing research on the cancers of greatest relevance	(30)
Ristic et al.	The role of fascin-1 in the development, diagnosis and treatment of five gastrointestinal tumors was collated	review	Fascin-1 is a promising diagnostic marker	(31)
Gupta et al.	Regulatory mechanisms of fascin-1 expression Role of fascin-1 in gynecological cancers	review	To further define the role of fascin-1 targeting as a potential therapeutic route for gynecological cancer by establishing an animal model	(32)
Lin et al.	Biochemical and structural basis of fascin activation Mechanisms of fascin up-regulation of expression and transcriptional regulation Mechanisms of fascin effects on cancer cells	review	Find out more about fascin’s new features	(33)
Lamb et al.	The structure of fascin protein and the expression pattern and function Non-standardized functions of fascin proteins Regulatory mechanisms of fascin	review	Further clarification of the mechanisms by which fascin proteins exert non-standardized functions	(34)
Lamptey et al.	The role of fascin in tumorigenesis and embryo implantation	review	Study the effect of fascin on trophoblast transcription and metabolism	(35)

The up-regulation expression and mechanism of fascin-1 in a variety of cancers were analyzed by Hashimoto et al. (9, 16). They revealed that fascin-1 was expressed in a certain percentage of primary tumors of all tumors (9, 16, 21), most significantly in aggressive pancreatic tumors and non-small cell lung cancer (9). Meanwhile, up regulation of fascin-1 expression could enhance the proliferative activity of cancer cells and promote the formation of cell protrusions, thereby facilitating cell migration (9, 21). Immunohistochemical studies on cancer specimens showed that high expression of fascin-1 was associated with reduced overall survival rate and increased invasiveness, among other parameters (16, 21). The results of a MeTa-analysis by Tan et al. showed that fascin-1 was associated with increased mortality in colorectal, esophageal, and breast cancers and metastasis in gastric and colorectal cancers, but lymph node metastasis of esophageal or lung cancers was not associated with fascin-1 (30). The novel roles of fascin-1 in the pathogenesis, diagnosis, and management of gastrointestinal tumors and gynecological tumors were analyzed by Ristic et al. and Gupta et al. (31, 32). Fascin-1 expression was increased in esophageal squamous cell carcinoma (ESCC), gastric, colorectal, ovarian, uterine, and cervical cancers, and high levels of fascin-1 were related to clinicopathological parameters such as lymph node infiltration, distant metastasis, and reduced survival (31, 32).

The mechanisms of fascin-1 overexpression and promotion of tumor metastasis were elucidated in the article by Lin et al. (33). They concluded that the overexpression of fascin-1 in cancer is unlikely to be due to epigenetic regulation, but rather to activation of NF-κB and JAK-STAT signaling by inflammatory factors in an environment of hypoxia and nutrient deficiency in inflammation (21, 33). For the mechanism by which fascin-1 promotes tumor metastasis, it promotes tumor cell migration by coordinating cell membrane protrusion and cell adhesion on the one hand, and metastatic colonization by promoting capillary extravasation to the mesothelial cell layer on the other (33). Meanwhile, fascin-1 can control metastatic colonization by controlling mitochondrial F-actin and mitochondrial metabolism (33). Fascin-1 participates in the regulation of pivotal oncogenic pathways, such as MAPK, Wnt/β-linked protein, PI3K/AKT, EMT, etc (21).

In addition to the role of fascin-1 in tumors, fascin-1 has been found to be associated with many neurological-related diseases, such as absence seizures, epilepsy, and down syndrome (16, 34). The review by Lamb et al. focused on the various functions of fascin-1 and elucidated that fascin-1, in addition to its bundled actin functions, also had many non-standard functions (34). For example, it interacts with the Linker of the Nucleoskeleton and Cytoskeleton (LINC) Complex, binds to microtubules and regulates actin binding protein activity and mitochondrial function (34). Meanwhile, the review by Lamptey et al. also compiled an analysis of the role of fascin-1 during human embryogenesis (35). It was found to regulate the epithelial-mesenchymal transition in placental formation and early embryogenesis and to achieve homeostasis (35).

Other people still have great expectations for future studies of fascin-1 (9, 16, 21, 30–35). Meanwhile, the results suggest that fascin-1 is a promising marker for tumor diagnosis and prognosis (9, 16, 30–32), but further studies are still needed to test the therapeutic potential of this protein (21, 31).

## Fascin-1 and non-small cell lung cancer

### Expression of fascin-1 in NSCLC and its potential as a prognostic marker in NSCLC

According to the latest statistical analysis of cancer, lung cancer has the highest mortality of all cancers, of which the five-year survival rate is no more than 22% (1), and the main reason for its low survival rate is the occurrence of distal metastasis (36). Non-small cell lung cancer (NSCLC) accounts for more than 85% of the total number of lung cancer (36). Among the actin-binding protein family, fascin-1 has the greatest impact on distant metastasis in NSCLC (37). Recent studies have found that the expression level of fascin-1 is considerably higher in NSCLC than in normal lung tissue (**Table 2**). The expression level of fascin-1 is closely related to tumor invasion and metastasis, which affects the survival time of tumor patients (38–40) (**Table 2**). This is an important reason why fascin-1 is expected to be a prognostic marker for NSCLC.

**Table 2:** Relationship between high expression of fascin-1 and clinical parameters in respiratory related cancers.

					Relevance Between High Fascin-1 Expression and:						
Type of Cancer	Specimen	Sample size	Methods	Lymph Node Metastasis	Distant Metastasis	Reduced Survival	Clinical stage	Other Outcomes	Correlation degree	Ref.
NSCLC	tissue	220	IHC	unk	+	+	+	Ki-67 labelling index	+	(38)
	tissue	49	IHC	+	unk	unk	+	lymphovascular invasion	+	(39)
								age, gender, tumor size histological subtype	−
	tissue	98	IHC	+	unk	unk	unk	Ki-67 labelling index TNM stage	+	(40)
								gender, age, histological type	−
	tissue	46	qPCR	+	+	unk	unk	unk	unk	(37)
	plasma	154	ELISA	unk	unk	unk	unk	relapse	+	(41)
	tissue	81	IHC	+	unk	+	+	poor prognosis	+	(42)
								age, gender, tumor size tumor grading pleural effusion	−
	plasma	110	ELISA	unk	unk	unk	+	age, gender smoking history primary sites differentiated degree	−	(43)
	tissue	84	IHC	+	unk	+	+	age groups	+	(44)
								sexes, T staging pathological classifications	−
	tissue	61	IHC	+	unk	unk	−	tumor diameter	+	(45)
								gender, age differentiated degree pathological stage pleural effusion	−
	tissue	unk	IHC	+	unk	+	unk	tumor size advanced staging	+	(46)
	tissue	128	IHC WB qPCR	+	unk	+	unk	TNM stage	+	(47)
								age, sex, tumor size differentiated degree smoking history	−
	plasma	501	ELISA	+	+	+	unk	gender smoking history histological subtype T status, N status M status, TNM stage	+	(48)
								age, tumor size	−
	plasma	156	RT-PCR WB IHC	+	+	+	+	differentiated degree N classification M classification	+	(49)
								age, gender smoking T classification	−
LSCC	plasma	150	IHC	+	unk	+	unk	T-stage histological grade tumor recurrence	+	(50)
								age, sex	−
	plasma	216	RT-PCR WB IHC	+	−	+	+	primary sites histologic differentiation poor tumor differentiation poor prognosis smoking status	+	(51)
								age, sex, metastasis	−
	plasma	30	IHC	unk	unk	unk	+	tumor stage, node stage	+	(52)
								age, sex, location	−
	plasma	40	qRT-PCR WB IHC	+	−	+	+	age, T staging N status, differentiation primary cancer site smoking status	+	(53)
								sex	−
OSCC	plasma	40	IHC	+	unk	+	unk	tumor staging, tumor size differentiation	+	(54)
								age, sex	−
	plasma	46	RT-PCR IHC	+	unk	+	unk	tumor recurrence	+	(55)
								age, sex, differentiation	−
	plasma	129	IHC	+	+	unk	+	Size, histological grading	+	(56)
								age, sex, location	−
	plasma	131	IHC IF	+	unk	+	unk	tumor stage differentiation tumor recurrence poor prognosis	+	(57)
				age, sex, location tumor size				−

* [unk] = unknown; [+] = enhanced; [−] = no effect.

Furthermore, the results of several studies on tumor tissues showed that elevated fascin-1 RNA and protein levels were significantly relevant to lymph node metastasis and TNM stage, but not with age, gender, tumor size or differentiation (**Table 2**). The expression level of fascin-1 was significantly inversely proportional to the survival time of NSCLC patients (**Table 2**). The measurement of fascin-1 concentrations in NSCLC patients’ serum revealed that with the increase of fascin-1 expression in patients’ serum, the tumor is prone to metastasis and indicates a poor prognosis (41, 48). Based on these findings, fascin-1 levels, both in tissues and in serum, are considered promising as new targets and prognostic indicators for assessing the prognosis of NSCLC patients. Although these studies suggest that fascin-1 is a promising prognostic marker in NSCLC, further studies are needed to determine whether it has therapeutic potential.

### The role and mechanism of fascin-1 in NSCLC

**Figure 2:**
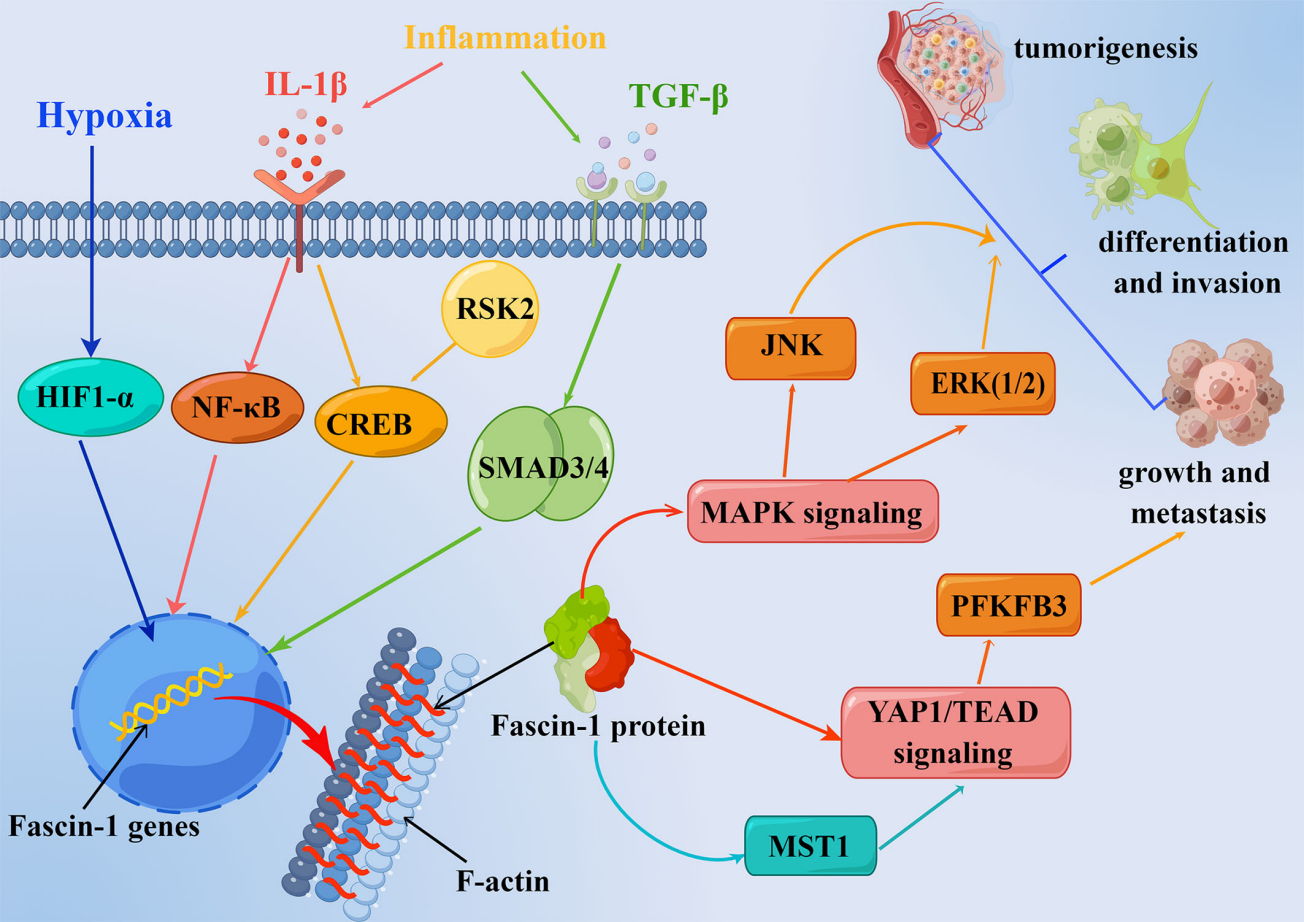
Molecular regulation mechanism of fascin-1 overexpression in respiratory related cancers. SMAD3/4, CREB, NF-κB, HIF1-α and other transcription factors can be activated by inflammatory microenvironment factors (IL-1β, TGF-β) and hypoxia, so as to up regulate the transcription of fascin-1 protein. Fascin-1 protein can promote tumorigenesis, invasion and metastasis through MAPK, YAP1/TEAD and other signal pathways.

Fascin-1 overexpression promoted the development of NSCLC by boosting cell growth and metastasis (25). Moreover, silencing the expression of fascin-1 in NSCLC cell lines could inhibit the proliferation, invasion and metastasis of NSCLC cells (58). Experiments in mice showed that inhibition of fascin-1 function reduced the migration and metastasis of cancer cells (59). However, an *in vitro* and *in vivo* experiment showed that high expression of fascin-1 could improve the migration rate and invasion of tumor cells, but did not promote the growth of tumor nodules (40). One previous study found that fascin-1 promotes lung cancer metastasis and colonization by enhancing resistance to metabolic stress and promoting mitochondrial oxidative phosphorylation (46). Fascin-1 was found to activate PFKFB3 transcription through the YAP1/TEAD binding site in its promoter to further promote glycolysis in NSCLC cells, thereby promoting lung cancer cell metabolism and growth (60) (**Figure 2**). These experiments suggest that inhibition of fascin-1 may be a potential therapeutic strategy for the treatment of NSCLC, but further studies are still needed.

### Regulation of fascin-1 expression in non-small cell lung cancer

The mitogen-activated protein kinase (MAPK) pathway has been related to promoting tumor metastasis (**Figure 2**). The crucial role of the MAPK pathway in the development and progression of NSCLC has been demonstrated in previous studies (48, 61). By further detecting the expression and phosphorylation level of MAPK signaling molecules, it was found that the expression of fascin-1 could be down regulated by regulating MAPK pathway, so as to inhibit the metastasis and invasion of non-small cell lung cancer cells (58). Furthermore, by studying the YAP/TAZ signaling pathway, the core of the Hippo signaling pathway, fascin-1 was found to promote the growth and metastasis of non-small cell lung cancer cells by connecting with kinase MST1 and activating the transcriptional activity of YAP/TEAD complex (25). RSK2 is a Ser/Thr kinase that regulates cell proliferation, cell survival and cycle by phosphorylating cAMP response element binding (CREB) proteins (62–64). As a transcription factor, CREB participates in the signal pathway related to promoting tumor progression, stimulating growth, giving apoptosis resistance and promoting angiogenesis (65, 66). Li et al. found that the RSK2-CREB pathway can up-regulate expression of fascin-1 in lung cancer cell line A549, in clinical samples and in xenograft mouse models, thereby promoting cancer cell filopod formation and thus cancer cell invasion and metastasis (67). They concluded that fascin-1 is expected to become a prognostic marker of metastatic cancer, and RSK2-CREB-fascin-1 signaling pathway is expected to become a therapeutic target of metastatic cancer (67). At the same time, inflammation can promote TGF-β to induce fascin-1 overexpression through direct binding of the SMAD3-SMAD4 complex to the fascin-1 transcriptional start site (68).

A number of compounds were found to exert inhibitory effects on cancer cell invasion and metastasis by blocking the signaling pathway associated with fascin-1 (**Table 3**). The results *in vitro* and *in vivo* showed that G2, a pharmacological inhibitor of fascin-1, could significantly reduce the levels of PFKFB3 and YAP1 in lung cancer cells and nude mice (60). G2 can significantly inhibit the tumor growth on the lung of nude mice, which improves the survival rate of mice (60). And G2 can also significantly reduce the volume of tumor-like culture (60). Meanwhile, it was found that sevoflurane, a commonly used anesthetic, could reduce HIF-α levels by blocking the p38MAPK signaling pathway, further down-regulating fascin-1 expression and thus inhibiting the growth and metastasis of lung cancer cells (69, 70). These studies found the potential role of sevoflurane in lung cancer surgery and gave sevoflurane new clinical significance (69, 70). In another study, it was found that polyisoprene cysteine amide inhibitors (PCAIs) can induce the decrease of fascin-1 protein level in NSCLS cells, so as to inhibit F-actin tissue, filamentous foot and focal adhesions, and further reduce cell invasiveness (71).

**Table 3:** Inhibitory effects on the expression of fascin-1 of different compounds in NSCC and NPC.

Cancer Type	Compound	Experimental object	Outcomes	Refs.
NSCC	G2	H1650, A549 H292, H23, LLC	Inhibited cell growth and migration Inhibited the expression of YAP1 and PFKFB3	(60)
		BL6 mice, nude mice	Reduced the tumor burden Improved the survival	
	Sevoflurane	A549	Reduced Proliferation rate and hypoxia-induced migration ability Down-regulation of HIF-1α expression	(69)
	Sevoflurane	A549	Reduced Proliferation rate and migration ability Reducing the levels of p38 MAPK phosphorylation	(70)
	PCAIs	NCI-H1299	Decreased in cell invasion	(71)
NPC	Thiostrepton	C666-1, NP69	Repressed the migration ability	(72)

**Figure 3:**
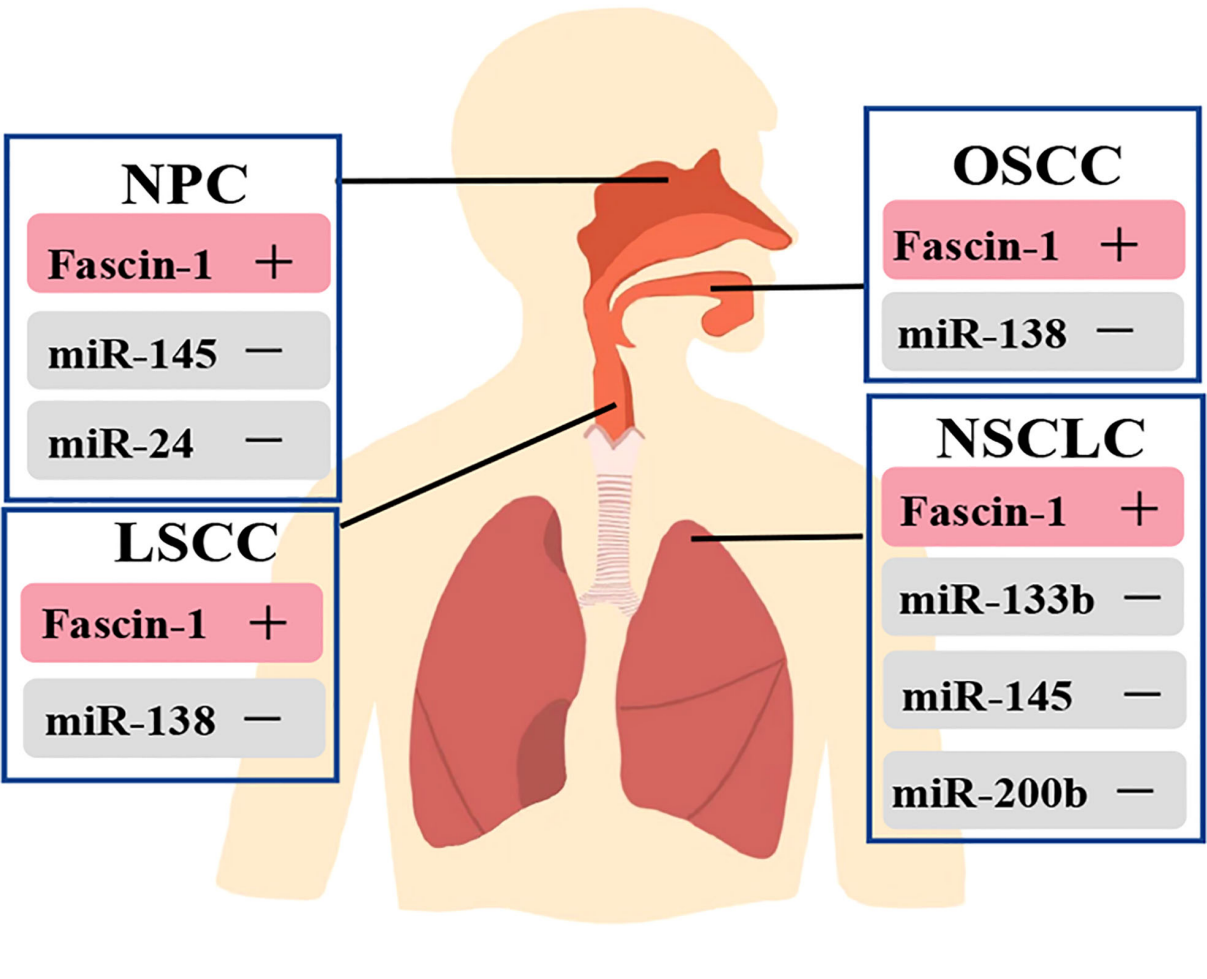
Relationship between miRNAs and regulation of fascin-1 expression in respiratory related tumors. Fascin-1 is highly expressed in respiratory related tumors, and activation of these miRNAs can inhibit the expression of fascin-1 in cancers.

Furthermore, microRNAs (miRNAs) regulate gene expression through post transcription, which is another mechanism to affect fascin-1 expression (34). In respiratory related cancers, down-regulation of these miRNAs leads to increase fascin-1 expression, which is associated with increased cell migration and invasion (34) (**Figure 3**). MiRNAs such as miR-145, miR-200b and miR-326 were labeled as potent repressors of fascin-1 (73–76). Thus, post-transcriptional regulation is one of the key mechanisms regulating fascin-1 expression, and activation of these miRNAs could serve as a potential therapeutic pathway.

## Fascin-1 and laryngeal squamous cell carcinoma

### Fascin-1 as a prognostic marker for LSCC

Apart from lung cancer, laryngeal squamous cell carcinoma (LSCC) of the larynx is the most usual cancer of the respiratory system (77). Immunostaining showed that fascin-1 was mainly distributed in the cytoplasm of tumor cells and the immune response to fascin-1 was homogeneous in the center of the tumor but enhanced at the tumor margin (50, 51). The expression of fascin-1 is significantly higher in LSCC than in adjacent normal margin tissue (51, 52, 78). Several studies have shown that fascin-1 expression in LSCC tissues is associated with T-stage, cervical lymphatic tract metastasis and clinical stage (**Table 2**). Also, high expression of fascin-1 was found to be associated with poor prognosis (51, 53, 79). To estimate the validity of fascin-1 expression as a prognostic marker in LSCC, it was found by using logistic regression models that fascin-1 could be an important independent predictor of LSCC recurrence (50). However, the results of another study showed that fascin-1 expression was not related to prognosis and that immunohistochemical studies were not helpful in predicting prognosis, and more emphasis should be placed on morphological findings (80). Based on these findings, most people believe that fascin-1 can be used as a potential molecular marker to judge the prognosis of LSCC (50, 51, 53, 79). Compared with other tumors, there are fewer studies on fascin-1 and LSCC, so further studies can be conducted to determine the potential of fascin-1 as a prognostic indicator for laryngeal cancer.

### The role of high expression of fascin-1 in LSCC

Based on the relationship between fascin-1 expression and clinical pathological prognostic parameters of LSCC, LSCC with high fascin-1 expression may be more aggressive than laryngeal carcinoma with low expression (52). Meanwhile, with the increase of fascin-1 level in LSCC patients, the tumor recurrence rate increased significantly, while the 3-year disease-free survival rate decreased significantly (50). In LSCC cells, by inhibiting the expression of fascin-1, it was found that the integrity of cytoskeleton structure was destroyed and the ability of cell migration decreased (81). Also, through in-depth bioinformatics analysis, fascin-1 may play a more important role in cancer progression than before (26).

### Regulation of fascin-1 expression in LSCC

In LSCC, aminoacyl tRNA synthetase complex interacting multifunctional protein 1 (AIMP1) and leukotriene A4 hydrolase (LTA4H) bind to and co-localize with fascin-1 (82). AIMP1 exerts its role in regulating cellular structure by binding to fascin-1 (82). Fascin-1 may be a substrate for LTA4H, and LTA4H may act by binding to fascin-1 binding to regulate the activity of fascin-1 to act (82). Meanwhile, knockdown of AIMP1 and LTA4H inhibits proliferation, migration, and invasion of LSCC cells (82). The results of another study showed that miR145-5p plays a key role in inhibiting LSCC progression through inhibition of fascin-1 (53). Luciferase analysis and xenograft model assay showed that miR-145-5p could inhibit the migration, invasion and growth of LSCC by downregulated the expression of fascin-1 gene (53). Therefore, they concluded that both miR-145-5p and fascin-1 are significant latent prognostic markers and therapeutic targets for LSCC (53).

## Fascin-1 and oral squamous cell carcinoma

### Results of fascin-1 overexpression in oral squamous cell carcinoma

It was observed that overexpression of fascin-1 can lead to the increase of F-actin structure such as filopodia and lamellipodia (57). According to the results of several studies, fascin-1 was found to be more expressed in human oral squamous carcinoma cells than in normal cells (54–56, 83, 84). Also, they found that high levels of fascin-1 expression had to do with lymph node metastasis and reduced disease-free survival (55, 57) (**Table 2**). Meanwhile, other studies found that the decreased expression of fascin-1 can reduce the migration and invasion of oral squamous cell carcinoma (OSCC) cells and increase cell adhesion (83–85). Overexpression of fascin-1 could promote cell proliferation (57). Also, univariate and multivariate survival analyses showed that fascin-1 expression levels could be an independent predictor of poor prognosis in OSCC (83).

Combined with the above findings, most believe that fascin-1 is linked to the poor prognosis of oral squamous carcinoma (**Table 2**), however, the results of one study revealed that although high expression of fascin-1 was present in most samples, it did not correlate significantly with patient prognosis (86).

### Mechanism of fascin-1 in OSCC

A known hallmark of aggressive tumor is the loss of E-cadherin expression, which results in reduced cell contact (87). Therefore, to assess the correlation between fascin-1 expression and E-cadherin, a negative correlation was found between fascin-1 and E-cadherin expression by detecting E-cadherin expression in OSCC specimens (55). Overexpression of fascin-1 is thought to enhance OSCC invasiveness by reducing the expression of E-cadherin (55). Furthermore, the mRNA expression levels of cathepsin B, cathepsin D, MMP-9 and MMP-10 were reduced, while the mRNA expression level of kinin release enzyme 5 (KLK5) was increased (88). This suggests that fascin-1 affects cancer invasion and progression by influencing the activity of matrix-degrading proteases (88). Furthermore, high expression of fascin-1 in OSCC derived cells leads to increased cell membrane protrusions, disruption of cell contacts and alterations in the actin cytoskeleton (57). Therefore, these data demonstrated that fascin-1 could be involved in OSCC invasion and progression through multiple pathways.

### Regulation of fascin-1 expression in OSCC

In the process of tumor invasion and progression, microenvironment plays a key role in the regulation of cancer cells (84). The study results showed that IL-1β is a key inducer of fascin-1 expression and can increase the invasiveness of oral cancer (84). Meanwhile, IL-1β by using ERK1/2 and JNK as intermediate signal molecules, NF-κB and CREB act as the signal pathway composed of transcription factors to induce the expression of fascin-1, which increases the invasiveness of cancer cells (84). Furthermore, Keratins 8 (K8) was found to be aberrantly expressed in squamous cell carcinoma (SCC) in previous studies, and its expression correlated with invasion and poor prognosis (89–92). Also, in the OSCC-derived cell line AW13516, knockout of the K8 gene leads to reduced fascin levels, resulting in altered actin organization and reduced cell migration (93). The results showed that there was a significant negative correlation between miR-138 and fascin-1 but not in miR-145. Meanwhile, miR-138 has the ability to target and regulate fascin-1 expression in OSCC, thus affecting the migration rate of cells (83) (**Figure 3**). Moreover, the results of study showed that fascin-1 activates AKT and MAPK pathways in OSCC-derived cells, thus promoting tumor progression (57). Therefore, fascin-1 is likely to be a new therapeutic target for human oral cancer (57).

## Fascin-1 and nasopharyngeal carcinoma

### Expression and role of fascin-1 in nasopharyngeal carcinoma

Nasopharyngeal carcinoma (NPC) is relatively rare compared to other cancers, but its global geographical distribution is highly uneven, with > 70% of new cases occurring in East and Southeast Asia (94). It is an Epstein-Barr virus-associated (EBA) malignancy and is the most usual head and neck cancer in China, mainly concentrated in the southern region (95). The role of fascin-1 in several human cancers has been confirmed through studies, but the number of studies on the presence and role of fascin-1 in NPC is relatively limited. In this context, immunohistochemical staining showed that the positive signal of fascin-1 was mainly concentrated in the cytoplasm (96). Fascin-1 was found to be overexpressed in NPC (78, 97, 98), and can participate in the progress of NPC by enhancing cell migration and adhesion (98).

### Regulation of fascin-1 expression in NPC

FoxM1 belongs to the fox transcription factor family FoxM1, which is a key factor involved in the regulation of the cell cycle from G1 to S phase, G2 to M phase and the transition to mitosis (99–101). FOXM1 has now been shown to be up-regulated in many human malignancies and to be a promising therapeutic target (44, 102–106). Thiostrepton, an inhibitor of FOXM1 (107), was found to significantly reduce the level of fascin-1 in C666-1 cells, thereby inhibiting the migration of NPC cells. However, because fascin-1 has not been reported as a direct or indirect target gene of FOXM1, the thiostrepton-FOXM1 pathway has not been established (72) (**Table 3**). Moreover, studies in NPC have identified miR-145 and miR-24 as inhibitors of fascin-1 expression, which were negatively correlated with fascin-1 expression (108) (**Figure 3**). MiR-145 and miR-24 were found to be significantly down-regulated in NPC cell lines and tissue samples, and that their ectopic expression could inhibit the growth and invasion of NPC cells by targeting fascin-1 (108, 109). Therefore, the miR-145-fascin-1 pathway and the miR-24-fascin-1 pathway may be potential new therapeutic targets for NPC patients (108, 109).

## Treatment potential and future directions

The treatment targets of fascin-1 in cancers mainly focused on small molecule inhibitors, miRNAs and inhibitory nanobodies.

Small-molecule inhibitors of fascin-1 reduce tumor cell migration and invasion (13, 59, 110–112). G2, a pharmacological inhibitor of fascin-1, significantly inhibited the growth of tumors and improved the survival rate of mice (60). In first-in-human clinical trial of ovarian cancer patients, NP-G2-044 (derivatives of G2) was safe and well tolerated (113). Also, compounds such as sevoflurane and polyisoprene cysteine amide inhibitors (PCAIs) can reduce the expression of fascin-1 (69–71). In non-respiratory related cancers, leucine aminopeptidase 3 (LAP3) inhibitors, migraine inhibitors could block fascin-1 activity (13, 110, 114).

Similarly, many miRNAs are tagged as potent post-transcriptional repressors of fascin-1 (**Figure 3**) by inhibiting cell proliferation, migration, and invasion (108, 115–117). Although it is possible to develop relevant therapeutic drugs based on miRNAs, there are still some limitations to using them in the clinical setting. To date, only 10 miRNA-based drugs have entered clinical trials, and none have reached Phase III (118). During clinical trials, the emergence of multiple immune-related side effects and severe hyperbilirubinemia forced the suspension of the trials (119–121). Targeting effects, routes of delivery, dosing issues, and drug delivery systems are the main challenges that must be overcome to develop miRNA-based cancer therapies (118).

*In vitro* experiments using inhibitory nanobody against fascin-1 protein in breast and prostate cancer cells have shown that they can inhibit invasion base formation and cell invasion (122). However, there are relatively few studies on inhibitory nanobody and no data on studies in respiratory related cancers. Therefore, there is a need to expand the research surface to determine the adaptability of inhibitory nanobodies in different tumors and also to determine whether they can be applied in clinical settings.

## Conclusions

Fascin-1 is overexpressed in a variety of human cancers, including respiratory related cancers, and is associated with several tumor clinicopathological parameters, such as increased tumor invasiveness, promotion of regional and distant metastasis, and reduced patient survival time. Meanwhile, fascin-1 is transcriptionally regulated by a variety of transcription factors (SMAD3/4, CREB, NF-κB, HIF1-α) and participates in a variety of cancer promoting signaling pathways, such as MAPK, YAP/TAZ, AKT, RSK2, etc. Therefore, fascin-1 is considered as a promising diagnostic marker and prognostic marker. Both *in vitro* and *in vivo* experiments have yielded good results on the therapeutic effects of fascin-1 as an anti-cancer target. Small molecular inhibitors, inhibitory nanobody and miRNAs have been found to potential therapeutic measure. Most studies focused on small molecule inhibitors and achieved good results. However, the development of anticancer drugs based on miRNAs still faces such major problems as targeting effect, drug delivery route and drug delivery system. The emerging inhibition nanobody is also a potential treatment method with good future prospects. At present, the study of the molecular regulatory mechanism of fascin-1 and its role with other proteins is still in its early stage, and we still have great expectations for the future study of fascin-1. Although most studies have shown a strong relationship between fascin-1 and the aggressive clinical course of multiple human cancers, most studies remain at the *in vitro* stage, lacking enough *in vivo* experiments to further prove the potential of fascin-1 for clinical application. A host of studies are still needed to determine whether fascin-1 can be used as a new biomarker and whether it exceeds the biomarkers currently in clinical.

## Author contributions

HY and NZ contributed to conception and design of the study. NZ wrote the first draft of the manuscript. YG organized the database. YG, QB, QW, YS and ZZ wrote sections of the manuscript. All authors contributed to the article and approved the submitted version.

## Funding

This research was funded by the Shandong Provincial Natural Science Foundation (grant numbers: ZR2020MH078 and ZR2020MH070), Shandong Province Medicine and Health Science and Technology Development Plan Project (grant number: 2019WS368), and College students' Innovative Entrepreneurial Training Plan Program of Jining Medical University (grant number: cx2021094).

## Acknowledgments

We made **Figures 1** and **2** by figdraw plotform. Thanks for the support of figdraw platform.

## Conflict of interest

The authors declare that the research was conducted in the absence of any commercial or financial relationships that could be construed as a potential conflict of interest.

## Publisher’s note

All claims expressed in this article are solely those of the authors and do not necessarily represent those of their affiliated organizations, or those of the publisher, the editors and the reviewers. Any product that may be evaluated in this article, or claim that may be made by its manufacturer, is not guaranteed or endorsed by the publisher.

## Abbreviations

ELISA, enzyme-linked immunosorbentassay; ESCC, esophageal squamous cell carcinoma; IF, immunofluorescence; IHC, immunohistochemistry; LSCC, laryngeal squamous cell carcinoma; NPC, nasopharyngeal carcinoma; NSCLC, non-small cell lung cancer; OSCC, oral squamous cell carcinoma; PCAIs, polyisoprenylated cysteinyl amide inhibitors; WB, western blot.
